# Catching a zebra in a surgical ward: atypical case of chylothorax caused by filariasis in a lupus patient—a case report from a surgical perspective

**DOI:** 10.1186/s13019-025-03778-z

**Published:** 2026-02-04

**Authors:** Euis Maryani, Arrayyan Muhammad, Ahmad Naufal Alfarisy, Rama Nusjirwan, Navy Laksmono, Mounti Martias, Prapanca Nugraha

**Affiliations:** 1 Division of Cardiovascular and Thoracic Surgery, Department of Surgery, Faculty of Medicine, Universitas Padjadjaran/Dr Hasan Sadikin General Hospital, 40161 Bandung, Indonesia; 2 Faculty of Medicine, Universitas Padjadjaran, Bandung, 40161 Indonesia; 3 Division of Digestive Surgery, Department of Surgery, Faculty of Medicine, Universitas Padjadjaran/Dr Hasan Sadikin General Hospital, Bandung, 40161 Indonesia

**Keywords:** Filariasis, Chylothorax, Lupus, Thoracotomy

## Abstract

**Background:**

Filariasis is a tropical disease caused by filariae, parasitic worms belonging to the phylum *Nematoda*. The clinical manifestations include asymptomatic microfilaremia, fever, lymphatic obstruction, and tropical eosinophilia. Chylothorax is the accumulation of chylous substances within the pleural space and is caused by thoracic duct damage or obstruction. Filariasis and systemic lupus erythematosus (SLE) have been identified as rare but potential causes of chylothorax.

**Case presentation:**

We reported a case of a 21-year-old North Sumatran female patient with SLE who presented in the surgery ward with a chief complaint of shortness of breath. On physical examination, dullness on palpation and diminished breath sound with rhonchi on auscultation were found over the lower two-thirds of the lung area. X-ray examination revealed pleural effusion in both lungs. The diagnosis of filariasis was confirmed via PCR and microscopic examination. Chylous pleural fluid was evacuated from both hemithoraxes following thoracotomy. The patient was also treated pharmacologically with bronchodilators, mucolytics, corticosteroids, and anthelminthics.

**Conclusion:**

This rare case of bilateral chylothorax caused by *Brugia malayi* in a patient with SLE highlights the need to consider filariasis in unexplained pleural effusions, especially in endemic regions. Early diagnosis and conservative antiparasitic therapy can yield excellent outcomes without surgical intervention.

## Introduction

Lymphatic filariasis (LF) is a mosquito-borne nematode infection caused mainly by Wuchereria bancrofti, Brugia malayi, and Brugia timori [1]. Its life cycle begins when infective third-stage (L3) larvae are deposited during a mosquito bite; the larvae mature into adult worms in lymphatic vessels, and mated females release microfilariae that are taken up by mosquitoes, perpetuating transmission [2]. Globally, LF affects over 120 million people in 72 countries, with roughly half of cases in South-East Asia. In Indonesia the burden is heterogeneous: a 2018 registry reported 10,681 chronic cases and 236 endemic districts, and a 2023 WHO review noted that, while 168 districts had stopped mass drug administration (MDA), 7,955 cases were reported in 2023 and 68 districts still required MDA [3, 4]. 

Clinical manifestations range from asymptomatic microfilaremia to chronic lymphatic dysfunction (lymphoedema, elephantiasis, hydrocele). Pulmonary involvement is uncommon but may present as chylothorax; filariasis can also cause tropical pulmonary eosinophilia (TPE), a hypersensitivity syndrome [2, 5]. Autoimmune serositis, most often caused by systemic lupus erythematosus (SLE), can independently produce or worsen pleural lymphatic leakage [6]. We report a unique case of bilateral chylothorax due to filariasis in an SLE patient, highlighting this atypical presentation in an endemic setting and underscoring the importance of clinicians making an accurate diagnosis and providing appropriate management.

## Case report

A 21-year-old North Sumatran female patient who had a history of shortness of breath since the past 2 weeks prior to admission was referred to the Department of Cardiothoracic Surgery of our hospital. The complaint worsened in the week before admission. The complaint was not relieved with rest, nor was it alleviated by changes in temperature or position. The patient also complained of swelling in both legs. There was no history of fever, cough, or respiratory wheezes.

One week prior to admission to our hospital, the patient was admitted to a regional lung hospital due to complaints of shortness of breath, fatigue, joint pain, facial flush, and photosensitivity. She was then diagnosed with systemic lupus erythematosus (SLE), hospitalized for 5 days and treated with corticosteroids. There was no history of hypertension, diabetes mellitus, or heart disease. There was also no such history or history of SLE among the patients’ family members.

On admission to our hospital, the patient was found to be fully conscious, with a blood pressure of 118/87 mmHg, pulse rate of 81 x/minute, respiratory rate of 26 x/minute, and blood oxygen saturation of 88% with 15 L per minute of oxygen through a nonrebreathing mask (NRM). On physical examination, dullness on percussion and diminished breath sounds with rhonchi on auscultation were found over the lower two-third of the lung area.

**Fig. 1:**
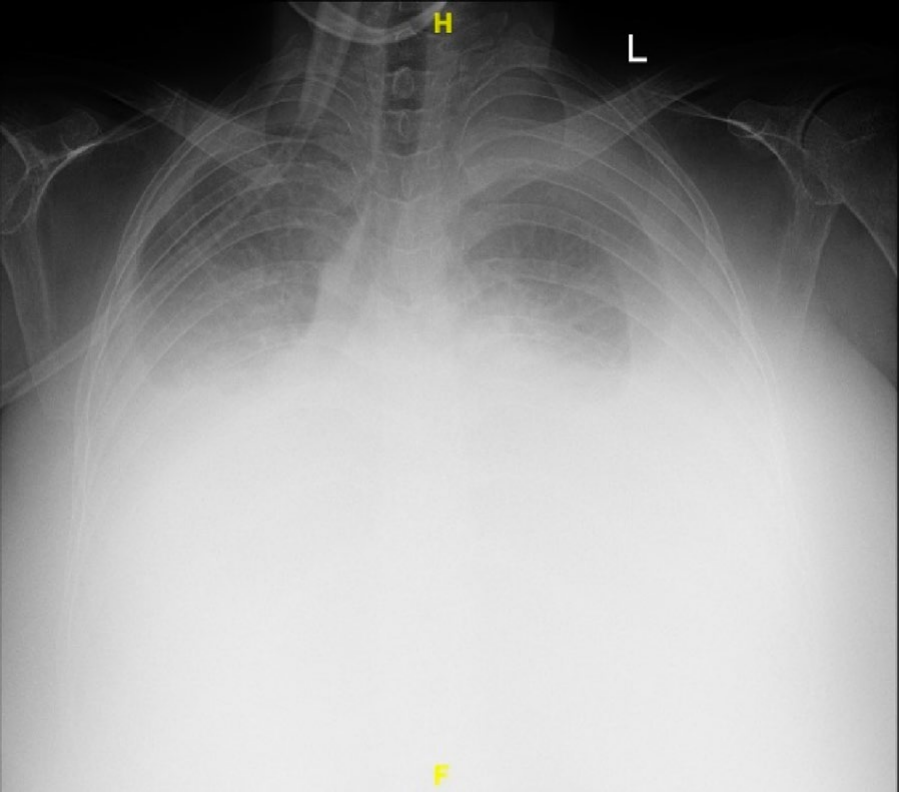
Bilateral pleural effusion was observed; homogenous opacification of both lower lung fields and blunting of the costophrenic angles, consistent with moderate to large fluid accumulation

Laboratory evaluation revealed a hemoglobin level of 13.6 g/dL, hematocrit of 41.1%, leukocyte count of 9.490 cells/mm^3^, thrombocyte count of 470.000 platelets/µl, albumin level of 2.7 g/dL, ureum level of 34.9 mg/dL, creatinine level of 0.51 mg/dL, sodium level of 140 mEq/L, potassium level of 3.9 mEq/L, and random blood sugar level of 89 mg/dL. Blood gas analysis revealed a pH of 7.358, pCO2 of 50.5, pO2 of 68.8, HCO3 of 28.7, BE of 3.1, and SaO2 of 91.9. Upon chest X-ray, pleural effusion was observed in both lungs (Fig. 1).

**Fig. 2:**
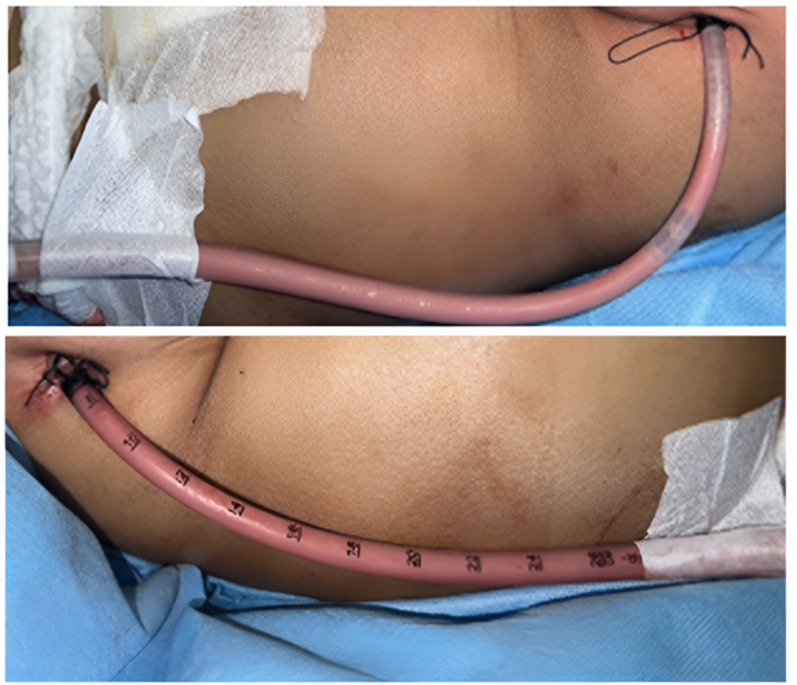
Pinkish-red chylous pleural fluid drained via thoracostomy tubes from both hemithoraxes, consistent with bilateral chylothorax. The appearance suggests lymphatic leakage, and the significant bilateral involvement indicates high-volume chyle accumulation

The patient underwent thoracotomy, which evacuated chylous pleural fluid from both hemithoraxes, the initial chylous drainage from each tube was 600 mL/24 h; no air bubbles were observed in the drain system and respiratory fluctuation (undulation) was positive (Fig. 2). Pleural fluid analysis showed: Rivalta positive, lactate dehydrogenase (LDH) 45.0 U/L, glucose 91 mg/dL, protein 2.900 mg/L, albumin 1.400 mg/L, 77 cells/µL, and triglyceride 610 mg/dL.

**Fig. 3:**
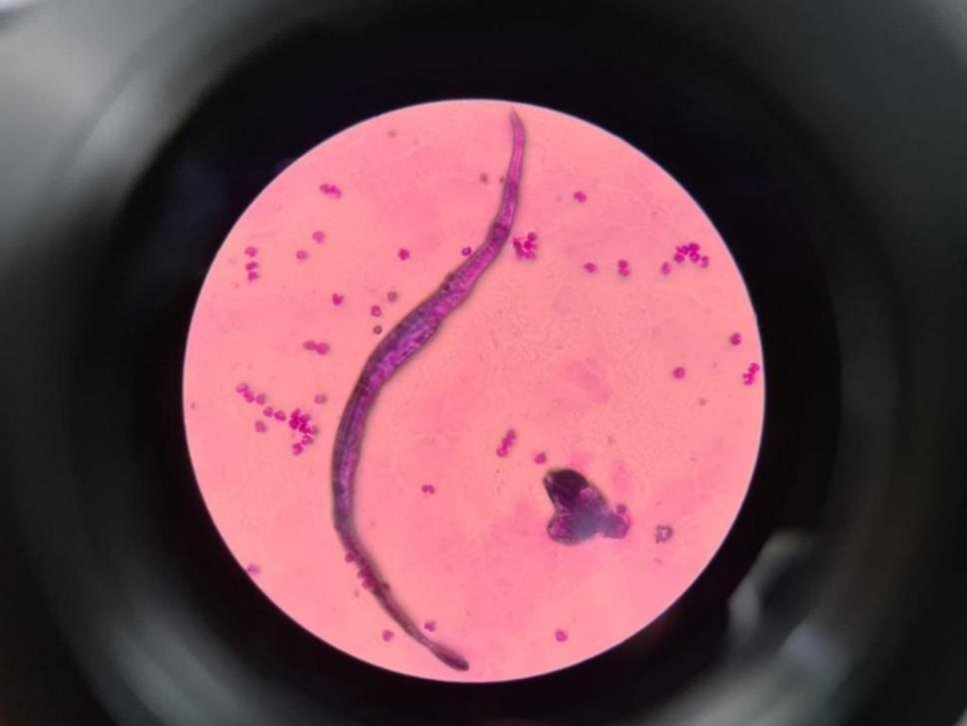
Microscopic examination of a peripheral blood smear revealed the presence of Brugia malayi microfilariae, identifiable by their sheathed body and nuclei that do not extend to the tip of the tail. Multiple surrounding lymphocytes and blood elements are also visible. These findings are consistent with lymphatic filariasis caused by Brugia malayi infection

Microscopic examination of Giemsa-stained thick and thin blood smears demonstrated microfilariae with morphologic features consistent with *Brugia malayi* (length, long headspace, characteristic terminal and subterminal tail nuclei and a pink-staining sheath) (Fig. 3). Species-specific PCR performed at a reference laboratory detected *Brugia malayi* DNA and confirmed the diagnosis. No serologic (ELISA) or rapid antigen tests (e.g., Filariasis Test Strip/Brugia Rapid) were performed.

The patient was treated pharmacologically with nebulized salbutamol q8h, n-acetylcysteine 400 mg three times a day, methylprednisolone 16 mg three times a day for 5 days, and a single dose of albendazole 400 mg and diethylcarbamazine (DEC) 350 mg. She was started on a medium-chain triglyceride (MCT) diet of 1,500 kcal/day and admitted to the Physical Medicine and Rehabilitation (PMR) department for chest physiotherapy.

**Fig. 4:**
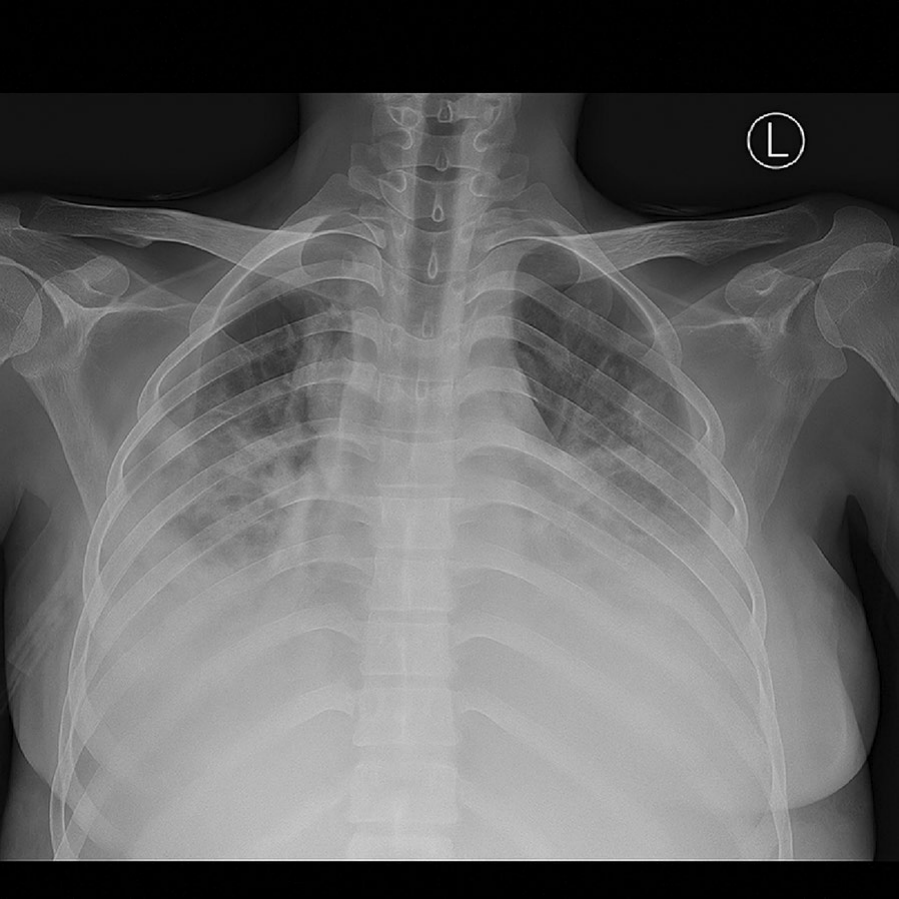
Follow-up chest X-ray showed a slight decrease in bilateral lung opacification, indicating partial resolution of pleural effusion. The lung fields appear better aerated. No new parenchymal infiltrates are observed

Upon follow up on day 5, the patient showed favorable response. Chylous drainage from both chest tubes had decreased to 100 mL/24 h. The complaint of shortness of breath subsided and blood oxygen saturation improved to 99% on 3 L per minute of oxygen. On physical examination rhonchi was no longer observed on both lung fields. Chest X-ray showed slight decrease in opacification of both lungs indicating decreased fluid accumulation in the pleural space (Fig. 4).

## Discussion

**Fig. 5:**
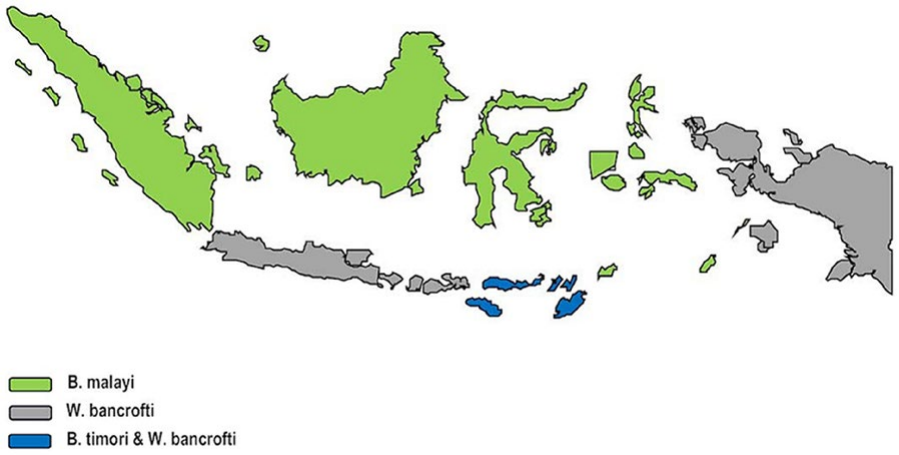
Geographic distribution of lymphatic filariae in Indonesia. B. malayi — Sumatra, Borneo, and Sulawesi; W. bancrofti — Java, Bali, West Nusa Tenggara, and Papua; co-endemic B. malayi and B. timori — East Nusa Tenggara and nearby islands of the Lesser Sunda chain [9]Reproduced from Tan M et al., PLOS Neglected Tropical Diseases (2014), under the Creative Commons Attribution (CC BY 4.0) license.

According to the World Health Organization (WHO), filariasis ranks as the second largest infectious disease vector after malaria. To date, more than 14,000 sufferers of elephantiasis have been found in 418 districts/cities throughout Indonesia, including 235 endemic districts/cities with a high risk of transmission [7, 8]. Indonesia harbors all three human lymphatic filariae, with *Brugia malayi* accounting for the majority of infections in many regions (Fig. 5); given the patient’s origin in North Sumatra and the local epidemiology, the morphologic identification of *B. malayi* is epidemiologically plausible [9].

Although PCR is considered the confirmatory standard for species identification, initial evaluation of stained blood smears remains a practical method to differentiate filarial species, as in this case. Key distinguishing morphologic features used to differentiate *B. malayi* from *W. bancrofti* included: (1) body length (*B. malayi* generally 177–230 μm vs. *W. bancrofti* 244–296 μm), (2) headspace length (longer in Brugia), (3) tail nuclei arrangement (terminal and subterminal nuclei separated by gaps in *B. malayi* but anucleate tail in *W. bancrofti*), and (4) sheath staining with Giemsa (pink in many *B. malayi* specimens versus colorless in *W. bancrofti*) [10, 11]. 

Tropical pulmonary eosinophilia (TPE) is a filarial hypersensitivity syndrome most often caused by filarial worms and should be considered in patients from endemic areas who present with nocturnal cough and dyspnea, very high eosinophil counts (often > 3,000/µL), markedly elevated IgE and antifilarial antibodies, characteristic pulmonary infiltrates on imaging, and a usually dramatic response to DEC [5, 12]. Our patient did not have the characteristic eosinophilic airway phenotype of TPE, but we have included it in the differential because it is an important, treatable filarial pulmonary manifestation in endemic regions.

The patient presented to the hospital with very heavy shortness of breath. Upon physical examination and chest X-ray, she developed bilateral pleural effusion. Surgical thoracocentesis should be performed on all patients who have more than a little pleural effusion (for example, more than 1 cm in height on lateral decubitus radiography, ultrasound, or CT) of unknown etiology. A diagnostic pleural tap, including a biochemical, cytological, and microbiological study of the fluid, is required for a proper diagnosis [13]. Our patient underwent chest tube thoracostomy insertion. The results of the pleural fluid examination revealed chylous fluid from both hemithoraxes, triglyceride level 610 mg/dL, and a positive Rivalta test. Although chylomicron testing is the confirmatory standard, it was not performed in this case. The markedly elevated triglyceride level, typical fluid appearance, and clinical context were considered sufficient to establish the diagnosis of chylothorax.

The causes of chylothorax can be divided into two categories: traumatic and nontraumatic. The most common cause, accounting for approximately 25–50% of cases, is trauma resulting from thoracic surgery. Other nontraumatic causes include obstruction of lymphatic outflow caused by malignancy, tuberculosis, or filariasis and, rarely, autoimmune diseases such as lupus [7]. Infection and SLE were our two main differentials for chylothorax at the beginning, as the patient met the EULAR/ACR diagnostic criteria for SLE; positive ANA test for the entry criterion and complaints of shortness of breath, joint pain, facial flush, and photosensitivity which together weighed more than 10 points in the criteria. [14] However, only 15 cases of SLE with chylothorax were reported from 2008-2019 [15], and the patient did not respond to corticosteroid treatment. The microbiology results revealed that *Brugia malayi* microfilaria was present in the blood.

SLE likely predisposes to non-traumatic chylothorax through inflammatory injury to lymphatic channels and serosal surfaces (lupus lymphangitis/serositis). Inflammation of lymphatic vessels increases lymphatic wall permeability, may raise endoluminal pressure and promote leakage or rupture of thoracic duct tributaries; when this inflammatory state is superimposed on filarial lymphatic obstruction, the combined effect (inflammation and mechanical obstruction by adult worms) markedly increases the risk of chyle extravasation into the pleural space. This mechanism is supported by case series and reviews describing SLE-associated lymphangitis/chylothorax and by reports in which glucocorticoids produced partial responses, consistent with an inflammatory contribution [6, 16, 17]. 

Pleural effusion is a rare manifestation of filariasis, with only 19 cases reported between 2000 and 2021 [18]. Filarial effusions usually have chylous characteristics because chyle leaks from the obstructed thoracic ducts. Nonchylous effusions resulting from microfilariae are uncommon. The filarial chyle is odorless and has a milky appearance; however, approximately half of the chyle is not milky [19]. This patient had a serosanguineous or pinkish-red chyle according to the results of the blood and serum mixture.

Chylothorax treatment involves conservative, radiologic, and surgical approaches. Conservative therapy includes dietary adjustments (low-fat diet), thoracocentesis for drainage, and addressing the underlying condition. Radiologic interventions include procedures such as stent shunt placement, lymphography, duct closure, and lymphatic pathway disruption. Surgery, which is indicated for rapid nutritional decline despite conservative measures, includes duct ligation and pleurodesis [20]. 

Surgery is generally not favored for nontraumatic chylothorax, and there is no consensus supporting routine surgical correction of thoracic duct defects. Because we confirmed a filarial etiology in this patient, we opted against further surgical intervention and pursued conservative management with albendazole plus DEC and supportive measures. This approach is consistent with WHO recommendations for managing lymphatic filariasis in areas without onchocerciasis [21]. 

The approach taken in this case has several shortcomings. For example, chest tube installation should be performed first at the previous hospital, followed by imaging examinations, which should be more complete, such as CT scans, to detect other possibilities, such as malignancy, and triglyceride analysis in the pleural fluid to confirm chylothorax.

## Conclusion

This case illustrates a rare occurrence of bilateral chylothorax caused by *Brugia malayi* infection in a patient with systemic lupus erythematosus (SLE). The coexistence of filarial lymphatic obstruction and lupus-related inflammation likely contributed to thoracic duct leakage. Diagnosis was confirmed through pleural fluid analysis and molecular testing. The patient showed favorable recovery with conservative pharmacologic therapy using diethylcarbamazine and albendazole. Clinicians in endemic regions should consider filariasis in unexplained chylothorax, particularly in patients with autoimmune disorders, to ensure timely and targeted management.

## Data Availability

The datasets used and/or analyzed during the current study are available from the corresponding author upon reasonable request.

## Abbreviations

SLE: Systemic lupus erythematosus
LF: Lymphatic Filariasis
PCR: Polymerase chain reaction
NRM: Non-rebreathing mask
TPE: Tropical pulmonary eosinophilia

## References

[1] Medeiros ZM, Vieira AVB, Xavier AT, Bezerra GSN, Lopes M, de Bonfim FC. Lymphatic filariasis: a systematic review on morbidity and its repercussions in countries in the Americas. Int J Environ Res Public Health. 2021;19(1):316. 35010576 10.3390/ijerph19010316 PMC8751179

[2] CDC - DPDx - Lymphatic Filariasis [Internet]. 2019 [cited 2025 Oct 7]. Available from: https://www.cdc.gov/dpdx/lymphaticfilariasis/index.html

[3] Aisyah DN, Kozlakidis Z, Diva H, Trimizi SN, Sianipar LR, Wijayanti E et al. The Spatial-Temporal distribution of chronic lymphatic filariasis in indonesia: A 18-Year Registry-Based analysis. Microbiol Res 2022 Sept 25;13(4):681–90.

[4] Indonesia launches final. push to eliminate lymphatic filariasis, leprosy and yaws [Internet]. [cited 2025 Oct 7]. Available from: https://www.who.int/indonesia/news/detail/10-10-2024-indonesia-launches-final-push-to-eliminate-lymphatic-filariasis--leprosy-and-yaws

[5] Jha SK, Killeen RB, Mahajan K. Tropical Pulmonary Eosinophilia. In: StatPearls [Internet]. Treasure Island (FL): StatPearls Publishing; 2025 [cited 2025 Oct 7]. Available from: http://www.ncbi.nlm.nih.gov/books/NBK557524/32491456

[6] Masnammany A. Refractory chylothorax ; a rare presentation of Systemic Lupus Erythematosus ( SLE ). Worldw Med. 2019;0:1. 10.5455/ww.59800.

[7] Meliyanie G, Andiarsa D, Program Eliminasi Lymphatic Filariasis di Indonesia. 2019 May 18 [cited 2025 Oct 7];3. Available from: https://ejournal2.litbang.kemkes.go.id/index.php/jhecds/article/view/1790

[8] Susanto I. Buku Ajar parasitologi Kedokteran. 4th ed. Jakarta: Badan Penerbit Fakultas Kedokteran Universitas Indonesia; 2017.

[9] Tan M, Kusriastuti R, Savioli L, Hotez PJ. Indonesia: an emerging market economy beset by neglected tropical diseases (NTDs). PLoS Negl Trop Dis. 2014;8(2):e2449. 24587452 10.1371/journal.pntd.0002449 PMC3937254

[10] Mathison BA, Couturier MR, Pritt BS. Diagnostic identification and differentiation of microfilariae. J Clin Microbiol. 2019;57(10):e00706–19. 31340993 10.1128/JCM.00706-19 PMC6760958

[11] Diagnosis. and treatment [Internet]. [cited 2025 Oct 7]. Available from: https://www.who.int/teams/control-of-neglected-tropical-diseases/lymphatic-filariasis/diagnosis-and-treatment

[12] Mullerpattan JB, Udwadia ZF, Udwadia FE. Tropical pulmonary eosinophilia–a review. Indian J Med Res. 2013;138(3):295–302. 24135173 PMC3818591

[13] Schild HH, Strassburg CP, Welz A, Kalff J. Treatment Options in Patients With Chylothorax. Dtsch Ärztebl Int [Internet]. 2013 Nov 29 [cited 2025 Oct 7]; Available from: https://www.aerzteblatt.de/10.3238/arztebl.2013.0819 10.3238/arztebl.2013.0819 PMC3865492 24333368

[14] Aringer M, Costenbader K, Daikh D, Brinks R, Mosca M, Ramsey-Goldman R, et al. 2019 European league against Rheumatism/American college of rheumatology classification criteria for systemic lupus erythematosus. Ann Rheum Dis. 2019 Sept;78(9):1151–9. 10.1136/annrheumdis-2018-214819 31383717

[15] Zhang GH, Zhang LL, Wang YH, Shen WB. Clinical characteristics of systemic lupus erythematosus with chylothorax and/or chylous ascites: an analysis of 15 cases in China. Medicine. 2020;99(51):e23661. 33371102 10.1097/MD.0000000000023661 PMC7748198

[16] Barillas S, Rodas A, Ardebol J, Martí JL. Nontraumatic chylothorax secondary to lymphoma and filariasis. J Surg Case Rep. 2020 Sept 1;2020(9):rjaa309. 10.1093/jscr/rjaa309 PMC7502309 32983405

[17] Soysal DE, Hizar Turan S, Ozmen M, Pekdiker M, Kalender ME, Koc E, et al. A rare case of Systemic Lupus Erythematosus with chylous ascites and chylothorax. Case Reports in Rheumatology. 2013;2013:797696. 23864976 10.1155/2013/797696 PMC3705745

[18] Aggarwal P, Subramanian S, Saini V, Aggarwal D. Filariasis presenting as isolated pleural effusion: a case report and mini review. Trop Doct. 2021;51(1):111–4. 33108966 10.1177/0049475520964399

[19] Vadala R, Talwar D, Talwar D. Recurrent non-traumatic idiopathic chylothorax: a diagnostic dilemma with therapeutic challenge. Respirol Case Rep. 2020;8(7):e00637. 32884811 10.1002/rcr2.637 PMC7456609

[20] Ur Rehman K, Sivakumar P. Non-traumatic chylothorax: diagnostic and therapeutic strategies. Breathe. 2022;18(2):210163. 36337134 10.1183/20734735.0163-2021 PMC9584559

[21] Background. In: Guideline: Alternative Mass Drug Administration Regimens to Eliminate Lymphatic Filariasis [Internet]. World Health Organization; 2017 [cited 2025 Oct 7]. Available from: https://www.ncbi.nlm.nih.gov/books/NBK487832/29565523

